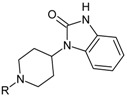# Current Concepts and Treatments of Schizophrenia

**DOI:** 10.3390/molecules23082087

**Published:** 2018-08-20

**Authors:** Piotr Stępnicki, Magda Kondej, Agnieszka A. Kaczor

**Affiliations:** 1 Department of Synthesis and Chemical Technology of Pharmaceutical Substances, Faculty of Pharmacy with Division of Medical Analytics, Medical University of Lublin, 4A Chodzki St., PL-20093 Lublin, Poland; piotr.stepnicki93@gmail.com (P.S.); magda.kondej@onet.pl (M.K.); 2 School of Pharmacy, University of Eastern Finland, Yliopistonranta 1, P.O. Box 1627, FI-70211 Kuopio, Finland

**Keywords:** antipsychotics, dopamine, drug design, drug targets, schizophrenia

## Abstract

Schizophrenia is a debilitating mental illness which involves three groups of symptoms, i.e., positive, negative and cognitive, and has major public health implications. According to various sources, it affects up to 1% of the population. The pathomechanism of schizophrenia is not fully understood and current antipsychotics are characterized by severe limitations. Firstly, these treatments are efficient for about half of patients only. Secondly, they ameliorate mainly positive symptoms (e.g., hallucinations and thought disorders which are the core of the disease) but negative (e.g., flat affect and social withdrawal) and cognitive (e.g., learning and attention disorders) symptoms remain untreated. Thirdly, they involve severe neurological and metabolic side effects and may lead to sexual dysfunction or agranulocytosis (clozapine). It is generally agreed that the interactions of antipsychotics with various neurotransmitter receptors are responsible for their effects to treat schizophrenia symptoms. In particular, several G protein-coupled receptors (GPCRs), mainly dopamine, serotonin and adrenaline receptors, are traditional molecular targets for antipsychotics. Comprehensive research on GPCRs resulted in the exploration of novel important signaling mechanisms of GPCRs which are crucial for drug discovery: intentionally non-selective multi-target compounds, allosteric modulators, functionally selective compounds and receptor oligomerization. In this review, we cover current hypotheses of schizophrenia, involving different neurotransmitter systems, discuss available treatments and present novel concepts in schizophrenia and its treatment, involving mainly novel mechanisms of GPCRs signaling.

## 1. Introduction

Schizophrenia is an important health issue, affecting almost 1% of the population, frequently with significant social and economic implications, as patients often suffer from unemployment and are homeless. Moreover, antipsychotics prescribed to treat schizophrenia are used in bipolar affective disorder, which has a prevalence of 2.3% in the population. Consequently, about 16.5 million patients in the EU need antipsychotics on a daily basis. This generates a significant healthcare costs, as central nervous system (CNS) disorders are among the most costly medical conditions (EUR 386 billion annually in the EU) [1]. Current treatments of schizophrenia have significant limitations. Firstly, they are efficient for only about half of patients enabling them independent life [2]. Secondly, they ameliorate mainly positive symptoms (e.g., hallucinations and thought disorders which are the core of the disease) but negative (e.g., flat affect and social withdrawal) and cognitive (e.g., learning and attention disorders) symptoms remain untreated [3]. Thirdly, they involve severe neurological and metabolic side effects and may lead to sexual dysfunction or agranulocytosis (clozapine) [4]. The reason for only partial effectiveness of current antipsychotics is the pathomechanism of schizophrenia which is not adequately understood due to its complexity and involvement of many molecular targets.

The current understanding of schizophrenia is constituted by the dopaminergic hypothesis which denotes alterations of dopamine neurotransmission in the mesolimbic system responsible for positive symptoms and mesocortical pathway, causing negative symptoms, complemented by the glutamatergic hypothesis which considers changes in prefrontal neuronal connectivity involving glutamatergic neurotransmission at NMDA receptor [5]. In particular, increased presynaptic dopamine synthesis is relevant for the pathogenesis of schizophrenia [6]. The methods of treatment of schizophrenia are classified as the first (mainly dopamine D_2_ receptor antagonists), second (multi-target antagonists with greater antagonism at serotonin 5-HT_2A_ receptor than at dopamine D_2_ receptor) and third generation antipsychotics represented, e.g., by aripiprazole, brexpiprazole and cariprazine. Aripiprazole is a partial dopamine D_2_ receptor agonist in G_α_ pathway but it can display agonist, partial agonist or antagonist activity at dopamine D_2_ receptor upon different signaling readouts [7]. In particular it is an antagonist or a partial agonist for β-arrestin-2 signaling pathway [7].

As G protein-coupled receptors (GPCRs) are classical and well-validated targets for antipsychotics, the elaboration of concepts on the nature of GPCR signaling opens novel and unexplored possibilities for more effective and safer antipsychotics. GPCR functioning is conceptualized by the ternary complex model which involves activation of the receptor by an agonist and transmission of the signal to G protein. However, it was reported that many GPCR ligand display high degree of promiscuity which was considered a drawback in GPCR-oriented drug discovery [8]. In many complex diseases including schizophrenia, the single target drugs turned out a failure, whereas multi-target drugs are much more efficient [9]. Clozapine has a low nanomolar affinity to several aminergic GPCRs which reflexes the complex pathomechanism of the disease.

Another important breakthrough in the field was discovery that a specific receptor can couple to a few G proteins and can signal independently on G proteins by occurring in an ensemble of conformations which trigger interaction with biased ligands to downstream effectors. This phenomenon is termed the functional selectivity [10,11] and may lead to safer drugs thanks to selective modulation of one pathway over another one. In the field of dopamine D_2_ ligands as antipsychotics, it was found that many clinically useful drugs are antagonists of β-arrestin recruitment [12]. Contrarily, it was also reported that dopamine D_2_ receptor ligands which are antagonists of Gα_i/o_ pathway and agonists of β-arrestin pathway may display beneficial antipsychotic properties with diminished extrapyramidal unwanted effects in animal models [7]. 

Allosteric modulation of GPCRs has lately been a hot topic in GPCR-oriented drug discovery [13,14] as allosteric mode of action brings important advantages over orthosteric drugs: better receptor or even pathway selectivity and fewer side effects and ceiling effect reducing the risk of overdosage [15]. This approach has not yet been exploited for antipsychotics, however a positive allosteric modulator (PAM) of dopamine D_2_ receptor, a peptidomimetic PAOPA, was proven efficient in attenuating symptoms of schizophrenia in animal models [16]. Few small molecule negative allosteric modulators (NAMs) of dopamine D_2_ receptor are known (SB269,652 [17]; homocysteine and analogs [18]) and their usefulness in schizophrenia needs to be further evaluated.

Finally, targeting heterodimers of dopamine D_2_ receptor which are distinct pharmacological entities, in particular adenosine A_2A_–D_2_ and serotonin 5HT_2A_–D_2_ heterodimers with bivalent ligands, dimer-specific monovalent ligands, compounds causing ligand-induced dimerization and peptides, peptidomimetics or small molecules disrupting dimer interface may lead to better pharmaceutics with higher selectivity and tissue specificity [19,20]. Among compounds targeting these dimers only bivalent ligands (not drug-like due to high molecular weight) are relatively easy to design. Compounds from other groups are not known either for D_2_ dimers (dimer-specific monovalent ligands and ligands inducing dimerization) or in the whole GPCR family (small molecules disrupting dimer interface). It was only reported that peptides, corresponding to the dopamine D_2_ receptor transmembrane regions TMVI and TMVII, effectively dissociated the dimer [21].

In this review, we cover current hypotheses of schizophrenia, involving different neurotransmitter systems, discuss available treatments and present novel concepts in schizophrenia and its treatment, involving mainly novel mechanisms of GPCRs signaling.

## 2. Schizophrenia as a Complex Disease

### 2.1. Dopaminergic Hypothesis

The dopaminergic hypothesis of schizophrenia is the fundament of the investigation and treatment of schizophrenia [22]. The first version of this hypothesis stressed the role of the excess of dopamine but it was developed into an idea linking prefrontal hypodopaminergia and striatal hyperdopaminergia and then to the current aberrant salience hypothesis [22].

The dopaminergic hypothesis of schizophrenia was proposed for the first time in the 1960s when chlorpromazine was introduced as the first antipsychotic and proved to treat positive symptoms of the disease [23]. Subsequently, the discovery that amphetamine produces psychosis was another proof for a role of excessive dopamine in schizophrenia. It was thus proposed that the increased dopamine neurotransmission might be a reason of this disease. The advancement of novel antipsychotics was in accordance with the dopaminergic hypothesis of schizophrenia as it was observed that positive symptoms of the disease can be attenuated with dopamine receptor antagonists. However, some findings contradicted this hypothesis, e.g., clozapine, which is a very effective antipsychotic in patients with resistant schizophrenia, has rather low affinity to dopamine D_2_ receptors. Moreover, some patients with schizophrenia also have normal level of dopamine metabolites in cerebrospinal fluid or serum. These contradictions and novel findings from PET studies led Davis et al. [24] to propose that schizophrenia involves diminished frontal and increased striatal dopaminergic neurotransmission. Moreover, they linked the positive symptoms of the disease with the striatal dopamine D_2_ receptor overactivation resulting from hyperactive mesolimbic dopamine projections while negative and cognitive symptoms result from the prefrontal cortex dopamine D_1_ receptor hypostimulation due to diminished mesocortical dopamine projections [22,24]. Further reformulation of this hypothesis has been reported [25].

Nowadays, aberrant salience hypothesis of psychosis most commonly links the dopaminergic system with the symptoms of schizophrenia. It is based on the incentive salience hypothesis [26] which suggests that the mesolimbic dopaminergic neurotransmission is crucial in the attribution of salience which governs attention and affects decision making and functioning [22]. The aberrant salience hypothesis assumes that the attribution of salience is disturbed by excessive dopamine firing in psychotic episode, while, in healthy individuals, dopamine is responsible for mediating contextually appropriate saliences [27]. This revised version of dopaminergic hypothesis of schizophrenia may explain some clinical and pharmacological features of the disease, i.e., why the schizophrenia patients do not develop all symptoms of psychosis at once or why antipsychotics exert their therapeutic effects after weeks [22]. Moreover, it can also shed new light on the side effect of diminished motivation in patients with antipsychotics medication and on the recurrence of psychosis after drug withdrawal.

As mentioned above, the dopamine D_2_ receptor is a drug target for all drugs against schizophrenia currently present on the market. First- and second-generation antipsychotics are dopamine D_2_ receptor antagonists while third-generation drugs are partial agonists or biased ligands of this receptor. Many drugs applied to treat schizophrenia are antagonists of D_2_-like (D_2_, D_3_ and D_4_) dopamine receptor subtypes [28]. As dopamine receptor play a key role in coordination of movement, memory and cognition, emotion and affect, and the regulation of prolactin secretion, blockade D_2_-like receptors may result in side effects linked with the long-lasting antipsychotics medication. This involves parkinsonian-like extrapyramidal symptoms typically resulting from the application of the first-generation antipsychotics and metabolic side effects (weight gain, hyperglycemia, increased risk of diabetes mellitus, dyslipidemia and gynecomastia) linked with the second-generation antipsychotics [28]. In this regard, there are some reports which indicate that D_3_ versus D_2_ dopamine receptor selective ligands may be an interesting alternative to treat schizophrenia [28]. It has also been found that the antagonism of dopamine D_3_ receptor may be partially responsible for blonanserin-caused cortical dopamine and acetylcholine efflux and cognitive improvement [29]. Importantly, selective dopamine D_3_ receptor antagonists are not efficient in antipsychotic animal models based on D_2_ receptor antagonism [30]. On the other hand, selective D_3_ receptor antagonists influence dopaminergic neurons electrical activity in the ventral tegmental area in the way characteristic for the second-generation antipsychotics, neutralize NMDA glutamate receptor blockade effects, and increase cortical dopamine and acetylcholine in microdialysis [30]. Contrary to dopamine D_2_ receptor antagonists, D_3_ antagonists beneficially affect several cognitive and social features in animal models, e.g., cognitive flexibility and executive function, that are deteriorated in patients with schizophrenia [30]. 

It was also demonstrated that prolonged dopamine D_2_ receptor blockade leads to downregulation of D_1_ receptors in the prefrontal cortex and, consequently, results in significant deterioration of working memory [31]. Thus, agonism at D_1_ receptors in the prefrontal cortex can have a key role in working memory and thus D_1_ receptor might be a target of choice for treating cognitive deficits in schizophrenia [32].

It should be stressed that, despite a key role of dopamine in the pathomechanism and clinical practice of schizophrenia, dopamine allows understanding the pathophysiology of the disease but not the reason per se [22]. In this context, dopamine functions as the common final pathway for a number of contributing environmental and/or genetic factors [22]. Thus, other neurotransmitters, in particular glutamate, are important for the pathomechanism of schizophrenia.

### 2.2. Glutamatergic Hypothesis

Glutamate belongs to the main excitatory neurotransmitters and is the most common neurotransmitter in the mammalian brain [33]. Glutamatergic pathways linking to the cortex, the limbic system, and the thalamus regions are important in schizophrenia [34,35]. Disturbances in the glutamatergic neurotransmission may influence synaptic plasticity and cortical microcircuitry, especially NMDA receptor functioning [36]. NMDA receptors belong to ligand-gated ion channels, and are important for excitatory neurotransmission, excitotoxicity and plasticity [37,38]. NMDA-receptor antagonists, e.g., phencyclidine and ketamine, can mimic psychosis with similar symptoms as in schizophrenia [39]. Moreover, in therapeutic trials substances which increase NMDA receptor signaling were reported to attenuate some symptoms in patients with schizophrenia [40]. Next, in postmortem studies, some disturbances in glutamatergic receptor density and subunit composition in the prefrontal cortex, thalamus, and temporal lobe were found [38,39,40] and these are brain regions with distorted stimulation while cognitive actions are performed by schizophrenia patients [41,42,43,44]. NMDA-receptor hypofunction state can lead to morphological and structural brain changes which can result in the development of psychosis [45,46]. It was hypothesized that levels of glutamate lower with age in healthy people, but it was not determined how they are affected by the chronic illness [47]. 

Antipsychotics may influence glutamate transmission by affecting the release of glutamate, by interaction with glutamatergic receptors, or by changing the density or subunit composition of glutamatergic receptors [35]. It was demonstrated that antipsychotics interacting with dopamine D_2_ receptor enhance the phosphorylation of the NR1 subunit of the NMDA receptor, thus reinforce its activation and consequent gene expression [48]. In this context, dopamine–glutamate interactions occur intraneuronally and intrasynaptically. There are also reports that action of some second-generation antipsychotics on NMDA receptors might be different from the effect of the first generation antipsychotics on this receptor [49]. Antipsychotics also influence glutamate transmission by acting on serotonin receptors [50].

Disturbances in glutamate signaling may be an attractive drug target for schizophrenia due to its key role in the pathomechanism of this disease in terms of cognitive impairment and negative symptoms [34,35]. Findings for hypoactivity of NMDA receptors in schizophrenia stimulated the clinical trials with substances activating this receptor [35]. Classical agonists at the NMDA are not useful here as excessive stimulation of NMDA receptors results in excitotoxicity and neuron damage. The glycine modulatory binding pocket on the NMDA receptor can be considered a more promising target. Similarly, positive allosteric modulators of another family of ionotropic glutamatergic receptors, i.e., α-amino-3-hydroxy-5-methyl-4-isoxazolepropionic acid (AMPA) receptors [51,52] as well as positive allosteric modulators of metabotropic glutamatergic receptors [53], might be considered promising new treatments of schizophrenia in accordance with the glutamatergic hypothesis of this disease.

### 2.3. Serotoninergic Hypothesis of Schizophrenia

The serotonin hypothesis of schizophrenia is derived from the reports about the mechanism of action of the hallucinogenic drug lysergic acid diethylamide (LSD) and its linkage to serotonin [54]. Consideration of the psychotic effects of LSD and the antipsychotic effects of, e.g., risperidone and clozapine, which are dopamine-serotonin receptor ligands, stimulated the research on connections between these neurotransmitters as a drug target in schizophrenia [55].

It was suggested that the overload of serotonin from the dorsal raphe nucleus (DRN) resulting from stress can disturb the activity of cortical neurons in schizophrenia [56]. Moreover, long-lasting extensive stress-derived serotonergic overload in the cerebral cortex in schizophrenia, in particular in the anterior cingulate cortex (ACC) and dorsolateral frontal lobe (DLFL), may be a key reason of this disorder [57].

Serotonin antagonists improve the extrapyramidal side effects of antipsychotics. Despite the lack of absolute proofs aberrance of serotonin signaling in the pathomechanism of schizophrenia, serotonin receptors, particularly 5-HT_3_ and 5-HT_6_, still represent promising drug targets for the discovery of novel multi-receptors antipsychotic agents which can alleviate cognitive and negative symptoms of the disease [58,59].

Serotonin-receptor-based signaling was proposed to have an important role in the action of the atypical antipsychotics [60]. It was suggested by Meltzer et al. [61] that significant 5-HT_2A_ receptor antagonism accompanied by diminished dopamine D_2_ receptor antagonism are the key pharmacological attributes which characterize clozapine and other second-generation antipsychotics and differentiate them from first-generation drugs. Several serotonin receptors, including 5-HT_2A_/_2C_, 5-HT_1A_, 5-HT_6_ and 5-HT_7_ receptors, can be partially responsible for the “atypicality” [62]. Many studies demonstrated that partial and full 5-HT_1A_ receptor agonists can diminish antipsychotic-induced catalepsy. Consequently, certain second-generation drugs which display a balance between dopamine D_2_ antagonism or partial agonism and 5-HT_1A_ receptor agonism/partial agonism result in low extrapyramidal side effects, which was demonstrated as low cataleptogenic activity in animal models [63]. Polymorphism of 5-HT_2C_ receptor gene is associated with olanzapine-induced weight gain [64]. Moreover, in meta-analyses, three genetic variants within serotonin genes were found linked to clozapine-associated weight gain: rs6313 and rs6314 within HTR2A gene and rs1062613 within HT3A gene [65]. Moreover, amisulpride, which has a high affinity for serotonin 5-HT_7_ receptors, reversed ketamine-induced social withdrawal in rat models [66]. Next, the antagonism of 5-HT_7_ receptors may be partially responsible for antidepressant and procognitive activity of amisulpride [67].

### 2.4. Other Aminergic GPCRs in Schizophrenia

Besides dopamine and serotonin receptors, other aminergic receptors are also linked to schizophrenia, i.e., histamine, muscarinic and adrenergic receptors. Histamine H_3_ receptor antagonists can be useful in treating cognitive deficits of schizophrenia [68].

Muscarinic receptors have a key role in modulating synaptic plasticity in the prefrontal cortex and stimulation of these receptors results in long-term depression at the hippocampo-prefrontal cortex synapse [69]. Cholinergic neurotransmission is impaired in patients with schizophrenia and in animal models of schizophrenia [69]. Importantly, muscarinic receptor antagonists deteriorate cognitive and negative symptoms in schizophrenia patients and xanomeline, a muscarinic receptor agonist, ameliorates all symptoms in schizophrenia patients and corresponding animal models [69]. 

There are also reports that α adrenergic receptors activity can be crucial for aberrant regulation of cognition, arousal, and valence systems associated with schizophrenia [70].

### 2.5. GABAergic Hypothesis of Schizophrenia

Gamma-aminobutyric acid (GABA) is the main inhibitory neurotransmitter in the CNS [71]. GABAergic interneurons are crucial for suppression of the CNS, key for the synchronization and oscillations of activity of neurons which are vital for perception, learning memory, and cognition [72]. GABA signaling disturbances cause imbalance between excitation and inhibition in the cerebral cortex which is one of the key factors in the pathomechanism of schizophrenia [73,74]. A role of GABA in schizophrenia was first noticed by Eugene Roberts in 1972 [75]. It was first suggested that GABA can be applied for the treatment of schizophrenia as it inhibits dopaminergic signaling, however recent evidence demonstrated that, in some models, GABA can have adverse effect on the dopamine activity [75].

Post-mortem studies supported the hypothesis about a changed GABA transmission in schizophrenia [72]. Importantly, the reduction of glutamic acid decarboxylase-67, GABA synthetic enzyme was observed in brain parts linked with critical cognitive functions (the dorsolateral prefrontal cortex, anterior cingulate cortex (ACC), motor cortex, visual cortex, and hippocampus) [72].

The decrease in transmission through the TrkB neurotrophin receptor results in a diminished GABA synthesis in parvalbumin-containing subpopulation of GABA neurons in the dorsolateral prefrontal cortex of schizophrenia patients. Despite both pro- and presynaptic compensative responses, the resulting change in the perisomatic inhibition of pyramidal neurons leads to a reduced capacity for the gamma-frequency synchronized neuronal functioning, which is necessary for the working memory functioning [76].

Changes in the GABA neurotransmission were found in basic and clinical research on schizophrenia and in animal models. The chandelier subtype of parvalbumin-positive GABA neurons can be particularly altered by, and characteristic for schizophrenia [77]. GABA interneurons are key to brain rhythm-generating networks, and synchrony of neural oscillations is crucial for the perception, memory and consciousness [78]. GABA signaling disturbances can result in changes in neural synchrony [78], abnormal gamma oscillations [79], and working memory deficits.

In clinical studies, the administration of GABA agonists was demonstrated to attenuate schizophrenia symptoms [80]. Nevertheless, it is not known how GABA interplays with other neurotransmitter systems which needs further investigation.

### 2.6. Nicotinic Receptors in Schizophrenia

Many people suffering from schizophrenia smoke. This can be attributed to the disease itself or its treatment [81]. There are numerous reports about disturbed brain cholinergic transmission in patients with schizophrenia [82]. Patients communicate that smoking helps them to relieve negative symptoms [83,84] which can be linked to their deficiencies regarding nicotinic receptors.

The high rate of smokers among patients with schizophrenia stimulated the research on the role of nicotinic receptors in this disorder [85]. Studying of α7 receptors with specific venomous toxins showed that α7 receptors are located in brain regions involved in cognition (e.g., the cortex and hippocampus) [85]. Deterioration of cognitive abilities such as working memory and cognitive flexibility, as well as attention, anticipate psychotic symptoms and are a prognosticator of functional outcome [85].

Preclinical and clinical research demonstrated that the diminished suppression of P50 auditory evoked potentials in schizophrenia patients can be linked with a lowered density of α7 nicotinic receptors in the CNS [86]. Schizophrenia patients display weak inhibition of P50-evoked responses to repeated auditory stimuli, which can result from damaged sensory gating. The influence of smoking, however, on the reversing of lowered auditory sensory gating in schizophrenia may be weakened as a result of the desensitization of the nicotine receptors. This was connected with the chromosome 15q14 locus of the α7 nicotinic receptor gene [87]. Consequently, nicotinic receptors can be an attractive drug target for the treatment of schizophrenia.

The results of trials with α7 nicotinic receptor agonists or positive allosteric modulators are promising [88] but require further investigation.

### 2.7. The Endocannabinoid System in Schizophrenia

The endocannabinoid system is changed in schizophrenia (i.e., elevated density of cannabinoid CB1 receptor binding in corticolimbic regions and increased levels of andamide in cerebrospinal fluid). This results in “cannabinoid hypothesis” of schizophrenia [89]. Moreover, certain genetic changes of the CNR1 gene may protect against schizophrenia or can promote a better pharmacological response to atypical antipsychotics [89].

### 2.8. Role of Inflammation and Oxidative Stress in the Pathomechanism of Schizophrenia

The role of inflammation and oxidative stress in schizophrenia is a focus of many studies [34]. It was reported that severe infections and immune disorders during the life-time are an additional risk factor for the development of schizophrenia [90,91]. Although prenatal infections alone do not seem to be a definitive risk factor, the neurodevelopmental exposure to infection can facilitate the occurrence of psychosis in offspring. This can be supported by the observation that during influenza epidemics women are more likely to give birth to children who develop schizophrenia [92]. In this regard, there are inflammatory models of psychotic disorders, e.g., the anti-NMDAR encephalitis syndrome [93]. In this disease, schizophrenia-liked symptoms are combined with elevated level of NMDA receptor autoantibodies. Immunotherapy is a treatment option for this syndrome. This is also indirect proof of involvement of glutamatergic system in the pathomechanism of schizophrenia.

Another treatable immune model of schizophrenia is gluten sensitivity with the occurrence of anti-tissue transglutaminase or anti-gliadin antibodies [94]. Indeed, there can be a possible relationship between diet rich in grain products with high gluten content and the occurrence or exacerbation of schizophrenia symptoms [95]. 

As a consequence of inflammation role in schizophrenia, antibiotics and anti-inflammatory agents have been tested to treat this disease but with a rather limited success [96]. However, a trial of 1000 mg per day of aspirin as add on treatment demonstrated improvements in the Positive and Negative Syndrome Scale (PANSS) total and positive symptoms [97].

The importance of oxidative stress in schizophrenia was suggested in the 1930s but it was for a long time underestimated. Recent studies indicate that the oxidative stress preferentially affects interneurons which can be subjected to antioxidant therapies [98,99]. Next, lipid-rich white matter is also sensitive to oxidative stress which can underlie myelin-associated deficiencies in schizophrenia [100].

## 3. Classical Approaches to Treat Schizophrenia

Due to poor understanding of the causes of schizophrenia, the treatment, engaging antipsychotic drugs, focuses mainly on reducing the symptoms of the disease. Although psychotic illnesses include a number of various disorders, the term antipsychotic drugs—also known as neuroleptics, major tranquillizers or anti-schizophrenic drugs—conventionally refers to drugs used to treat schizophrenia. The same drugs are also used to treat brain damage, mania, toxic delirium, agitated depression and other acute behavioral disturbances. In terms of pharmacology, most are antagonists of dopamine receptor, although many of them also have an affinity for other targets, especially serotonin receptors, which may have an impact on their clinical efficacy. Currently available drugs have many drawbacks when it comes to their efficacy and side effects. Even though gradual improvements with newer drugs have been achieved, radical new approaches require a deeper understanding of the pathomechanism and causes of the disorder that are still insufficiently understood [101].

The purpose of treatment is to reduce the suffering of the patient and to improve functioning in cognitive and social area. Life-long treatment with antipsychotic drugs is required in case of many patients. Neuroleptics relieve positive symptoms of schizophrenia such as thought disorder, delusions and hallucinations, and prevent relapse. Their effectiveness is lesser on negative symptoms, which include social withdrawal and apathy. In many patients, negative symptoms have a tendency to persist between periods of treated positive symptoms, but early begun treatment of schizophrenia may prevent the development of negative symptoms over time. Patients suffering from acute schizophrenia usually respond better to treatment than those with the symptoms of chronic disease. To prevent relapses, long-term treatment is usually necessary after the first episode of the disease. Doses that are effective in acute schizophrenia should ordinarily be continued as prophylaxis [102].

The clinical effectiveness of antipsychotics in enabling patients suffering from schizophrenia to lead relatively normal lives has been presented in many controlled trials. The patient population of psychiatric hospitals, which was comprised of mainly chronic schizophrenics, declined exponentially in the 1950s and 1960s. It took place due to the introduction of neuroleptics, as well as the changing professional and public attitudes in terms of hospitalization of mentally ill patients. However, antipsychotic drugs suffer severe limitations which include:

- (1) Some patients lack response to drug treatment. Clozapine is recommended in patients resistant to other neuroleptics. The 30% of patients that do not respond are classified as “treatment resistant” and represent a major problem regarding treatment. It is still unknown what underlies the difference between responsive and unresponsive patients, although there are some presumptions that polymorphisms within the dopamine and serotonin receptors family may be involved.
- (2) They are effective in relieving the positive symptoms (delusions, hallucinations, thought disorders, etc.) but most of them lack effectiveness in controlling the negative symptoms (social isolation, emotional flattening) and cognitive dysfunctions.
- (3) They may result in a wide range of side effects including extrapyramidal, sedative and endocrine effects that can limit patient compliance.
- (4) They may decline survival through pro-arrhythmic effects.

Antipsychotic drugs of second generation were believed to overcome these limitations to some degree. However, according to meta-analysis performed by Leucht and co-workers [103], only some of the examined second-generation neuroleptics, displayed better overall efficacy. Sudden cessation of administration of antipsychotic drugs may result in a rapid onset of psychotic episode, that are different from the underlying illness [101].

The antagonism of dopamine D_2_ receptors located in the mesolimbic pathway is believed to reduce the positive symptoms of schizophrenia. Unluckily, systemically administered neuroleptics do not distinguish between D_2_ receptors in different brain areas and thus D_2_ receptors present in other regions of the central nervous system will be blocked as well. As a result of this effect, antipsychotics lead to unwanted motor effects (blocking D_2_ receptors in the nigrostriatal pathway), enhanced secretion of prolactin (blocking D_2_ receptors in the tuberoinfundibular pathway), reduced pleasure (blocking D_2_ receptors in the reward system component in the mesolimbic pathway) and presumably they even exacerbate the negative symptoms of the disease (blocking D_2_ receptors located in the prefrontal cortex, although they occur in low abundance–D_1_ receptors are expressed at higher density). While all neuroleptics, excluding third generation, act by blocking D_2_ receptors and therefore, theoretically, should induce all of these side effects, some exhibit additional pharmacological activity (e.g., antagonism at muscarinic and 5-HT_2A_ receptor) that, to various degree, reduce unwanted effects. Blockade of 5-HT_2A_ receptor may also contribute to alleviating the negative and cognitive symptoms of schizophrenia [101]. 

The concept that serotonin dysfunction can be involved in the development of schizophrenia has come in and out of favor several times. As already mentioned, originally, it was based on the fact that LSD, which is a 5-HT_2A_ receptors partial agonist, produces hallucinations. However, nowadays, it is thought that serotonin is not directly associated with the pathogenesis of schizophrenia. Nonetheless, affecting serotonin receptors, combined with antagonism at D_2_ receptor, has resulted in development of new drugs with improved pharmacological and therapeutic profiles. These are serotonin 5-HT_2A_ and 5-HT_1A_ receptors that play an important role in the treatment of schizophrenia. 

5-HT_2A_ receptors belong to G_i_/G_o_-coupled receptors and, being activated, produce neuronal inhibition. In the nigrostriatal pathway, 5-HT_2A_ receptors control the dopamine release in this way. Drugs that are antagonists to 5-HT_2A_ (e.g., olanzapine, risperidone) enhance the release of dopamine in the striatum by decreasing the inhibitory effect of serotonin. It will manifest in reducing extrapyramidal side effects. Moreover, in the mesolimbic pathway, combined effects of antagonism at D_2_ and 5-HT_2A_ receptors are suggested to counteract the enhanced dopamine function that cause positive symptoms of schizophrenia. Furthermore, block of 5-HT_2A_ receptor appears to improve the negative symptoms on account of enhancing the release of both dopamine and glutamate in the mesocortical circuit. 5-HT_1A_ receptors, which are somatodendritic autoreceptors, inhibit serotonin release. Neuroleptics that are 5-HT_1A_ receptors agonists or partial agonists (e.g., quetiapine) may act by reducing the release of serotonin and thus increasing dopamine release in prefrontal cortex and the striatum.

Some phenothiazine antipsychotics (e.g., periyazine) have been proven to cause fewer extrapyramidal effects than others, which is thought to be correlated with their antagonist properties to muscarinic receptors. Some second-generation drugs (e.g., olanzapine) also exhibit muscarinic antagonist properties. Dopaminergic nerve terminals in the striatum are suggested to innervate cholinergic interneurons which express inhibitory D_2_ receptors [104]. Normally, there is an equilibrium between activation of dopamine D_2_ and muscarinic receptor. Antipsychotic drug that block D_2_ receptors in the striatum will lead to increased release of acetylcholine on to muscarinic receptors, producing extrapyramidal side effects, which are neutralized if the antagonist of D_2_ receptor also display antagonist activity at muscarinic receptors. Maintaining the balance between dopamine and acetylcholine was also the rationale behind the use of benztropine, the muscarinic antagonist, to reduce extrapyramidal side effects of neuroleptics. However, antagonist activity at muscarinic receptors may result in side effects such as blurred vision, dry mouth and constipation [101].

The term “atypical” is widely used, although it has not been clearly defined. In result, it refers to the diminished tendency of later drugs to cause motor side effects, but it is also used in describing compounds that have different pharmacological profile from first-generation antipsychotics. In practice, it frequently serves—not very usefully—to distinguish the large group of similar first-generation dopamine antagonists from the group of later compounds, which is characterized by higher degree of diversity. Distinction between first- and second-generation antipsychotic drugs rests on such criteria as: receptor profile, occurrence of extrapyramidal side effects (less in second-generation group), efficacy (especially of clozapine) in resistant to treatment group of patients, and efficacy against negative symptoms [101].

It is also worth mentioning that nowadays new system of nomenclature for psychotropic medications that is neuroscience-based nomenclature (and its further update neuroscience-based nomenclature-2) is recommended [105]. This system supplies a pharmacological driven nomenclature which focuses on pharmacology and mode of action, reflecting available knowledge and understanding about the targeted neurotransmitter, molecule, system being modified, and mode/mechanism of action. It also includes four additional dimensions: (1) approved indications; (2) efficacy and side effects; (3) “practical note” which summarizes the clinical knowledge that has been prioritized by “filtering” though the taskforce’s “opinion sieve”; and (4) neurobiology.

### 3.1. First-Generation Antipsychotics

The first-generation antipsychotic drugs act mainly by blocking dopamine D_2_ receptors in the brain. They do not exhibit a selectivity for any of the dopamine pathways in the central nervous system and therefore can lead to a range of side-effects, in particular extrapyramidal symptoms and elevated prolactin. In this and the following sections, the most common adverse effect of antipsychotics are mentioned.

The history of antipsychotic drugs dates to December 1950, when chlorpromazine, the first neuroleptic that belongs to the family of phenothiazines, was synthesized in France by the chemist Paul Charpentier as a result of research on new antihistaminic drugs. Further behavioral experiments confirmed antipsychotic properties of chlorpromazine. The release of chlorpromazine to the market took place in 1953 with the trade name *Largactil*, which derives from “arge” and “acti”, indicating the broad activity of the drug [106].

According to chemical structure the first-generation antipsychotics may be divided into several groups, as showed in Table 1 [107].

The prototypical and the largest group of antipsychotic drugs in terms of chemical structure is the group of phenothiazines. It may be divided into three subclasses, which comprise in total more than forty drugs. All of them share three-ring phenothiazine structure but differ with side chains joined to the nitrogen atom (position 10 of phenothiazine) and substituents in position 2, which affects the activity of the drug. The three subgroups of phenothiazines have been distinguished considering the side chain in position 10. They are aliphatic, piperidine, and piperazine phenothiazines. The potency of drugs depends on their side chain and therefore aliphatic and piperidine phenothiazines may be characterized as agents of low to medium potency, whereas the potency of piperazine phenothiazines is described as medium to high [108].

Aliphatic group of phenothiazine derivatives is generally characterized by explicit sedative effects and moderate extrapyramidal and antimuscarinic side effects. Piperidine phenothiazines have moderate sedative effects, but cause fewer extrapyramidal side effects than other groups. Drugs with side chain containing piperazine exhibit fewer sedative and antimuscarinic effects, but more pronounced extrapyramidal side effects than in case of aliphatic and piperidine phenothiazines [102].

Another group of typical antipsychotic drugs are butyrophenones (e.g., benperidol, droperidol, and haloperidol) whose pharmacological properties are similar to those of piperazine phenothiazines, although their antidopaminergic effect is probably higher than in the case of phenothiazines, with lower antihistaminic, antiadrenergic and anticholinergic effect. Thioxanthenes exhibit moderate sedative, extrapyramidal and antimuscarinic effects, whereas diphenylbutylpiperidines are characterized by reduced sedative, antimuscarinic and extrapyramidal effects [108].

The relative potency of typical antipsychotics can be expressed by comparison with chlorpromazine and according to that first-generation neuroleptics can be arranged from low to high potency. The measure of “chlorpromazine equivalence” defines the amount of the drug in mg which allows to achieve the same effect as administration of 100 mg of chlorpromazine. The examples of high potent antipsychotic drugs are haloperidol and fluphenazine, both with chlorpromazine equivalent dose of 2 mg. Thioridazine is an antipsychotic of low potency, according to this classification, and is comparable with chlorpromazine—it must be administered in the dose of 100 mg to achieve similar potency as 100 mg of chlorpromazine [101,108].

Due to affecting wide range of receptors and lack of selectivity to dopamine receptors located in the mesolimbic pathway, antipsychotic drugs result in numerous side effects. The most frequent and severe are extrapyramidal effects, such as dyskinesia, dystonias, akathisia, unwanted movements, muscle breakdown, tremors and rigidity, which occur as a result of blocking dopamine D_2_ receptors in the nigrostriatal pathway. High doses of typical antipsychotics may induce negative and cognitive symptoms by antagonism to dopamine receptors in the mesocortical pathway, whereas blocking those receptors in the tuberoinfundibular pathway increases the release of prolactin in the pituitary gland and leads to hyperprolactinemia. Another side effects on the CNS are sedation (resulting from antihistaminic activity), drowsiness, vertigo, disturbed sleep, agitation, nightmares, dementia, loss of memory, and depression. Blockade of α_1_ adrenergic receptors may cause hypotension. Side effects on cardiovascular system comprise also tachycardia, palpitation, arrhythmia or chest pain. Antipsychotic drugs affect also liver. They increase the concentration of alkaline aminotransferase in the serum and may cause side effects such as jaundice, reversible liver cell hyperplasia, necrosis and increased level of bilirubin. Some effects from the side of the urinary and reproductive system have also been reported: impotence, increased or decreased libido, priapism, polyuria, delayed and premature ejaculation, galactorrhea and anorgasmia. Side effects related to the gastrointestinal system, such as weight gain, nausea, dry mouth, heartburn, anorexia, epigastric distress, dyspepsia, constipation, increased level of pancreatic enzymes, and abdominal cramps, are also common. Other adverse reactions that may result from the treatment with antipsychotic drugs involve: hot flashes, nasal congestion, numbness, blurred vision, dry throat, neutropenia, leukopenia, agranulocytosis, chills, glaucoma, hyperlipidemia, and depression of the respiratory system [101,108].

### 3.2. Second-Generation Antipsychotics

The new era in treating schizophrenia has begun when, after almost forty years from the introduction of chlorpromazine, the first antipsychotic, FDA approved the clinical use of clozapine in the cases of treatment-resistant schizophrenia. Clozapine has been synthesized in the laboratories of Sandoz and, after clinical trials were performed, released to the market in such countries as Switzerland, Austria, West Germany and Finland. However, studies on clozapine performed at the same time in the USA resulted in reports of agranulocytosis leading to death in some patients treated with the drug. Thus, clozapine disappeared from the market for a long time, but the interest of the scientists in working on the drug did not diminish. Further studies proved the high efficacy of clozapine in the treatment-resistant forms of schizophrenia, which resulted in the FDA approval for the drug in this disease entity [109].

Clozapine was the first drug with a stronger ability to reduce negative symptoms and that causes fewer extrapyramidal symptoms than known to date antipsychotics. The discovery of clozapine contributed to the introduction of new drugs with more beneficial pharmacological profile than first generation antipsychotics. Generally, second=generation drugs, in comparison with classical antipsychotics, exhibit higher ability to block serotonin 5-HT_2A_ receptors than dopamine D_2_ receptors. Additionally, antagonism to D_2_ receptors is weaker in the case of second-generation antipsychotics comparing to those of first generation, which manifests in lower occurrence of extrapyramidal side effects. There are also hypotheses that suggest that atypical antipsychotics bind to dopamine receptors with high dissociation rates or that they are more likely to block dopamine receptors in cortical and limbic regions than in nigrostriatal pathway, which also contributes to lower risk of extrapyramidal effects [110]. 

**Figure 1:**
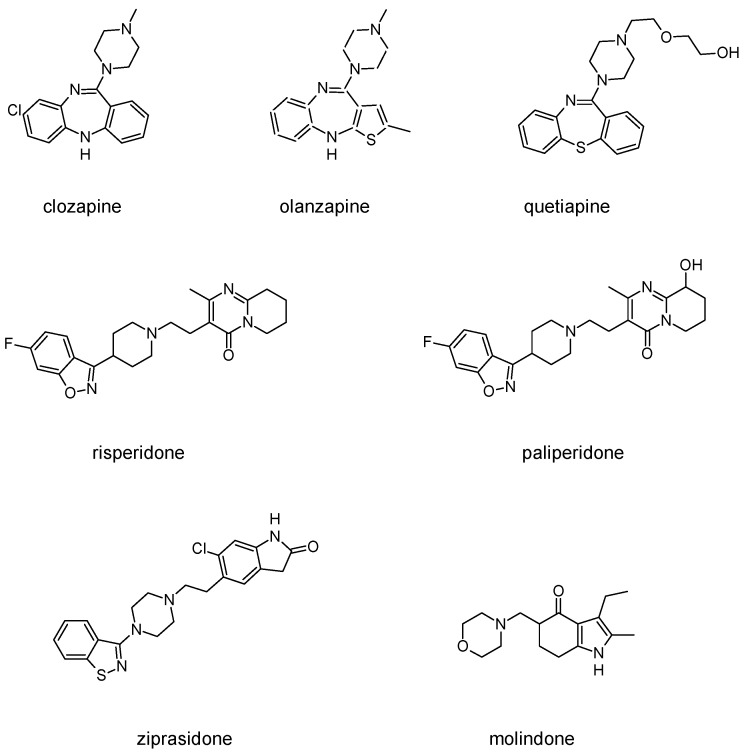
The chemical structures of representatives of second-generation antipsychotic drugs.

Besides schizophrenia, atypical antipsychotics are used in other diseases, such as bipolar disorder, anxiety disorder, obsessive-compulsive disorder, agitation associated with dementia and autism spectrum disorder [111]. This makes searching for novel antipsychotics even more important as there is a significant group of patients who need antipsychotics on a daily basis. Second-generation antipsychotics currently approved for clinical use include: clozapine, olanzapine, quetiapine, risperidone, paliperidone, ziprasidone, and molindone (Figure 1).

Clozapine, as an atypical antipsychotic, is dopamine and serotonin receptor antagonist. It binds to dopamine D_1–5_ receptors, with ten times higher affinity to D_4_ receptors than to D_2_. Both D_4_ and 5-HT_2A_ antagonist properties contributes to decrease of negative symptoms and lower occurrence of extrapyramidal side effects. The effect of clozapine on 5-HT_2A_ receptor signaling differs from classic GPCR antagonists, as it also induces 5-HT_2A_ receptor internalization and activates Akt signaling via a 5-HT_2A_ receptor-mediated event [112]. Thus, clozapine may also be considered a functionally selective agonist. At this serotonin receptor, clozapine is also serotonin 5-HT_1A_ receptor partial agonist, which is thought to have beneficial effect in terms of reducing negative and cognitive symptoms. Muscarinic receptors are also affected by clozapine: it blocks M_1_, M_2_, M_3_ and M_5_ receptors, while stimulates M_4_ receptor (which results in an excessive salivation as a side effect). Clozapine’s metabolite norclozapine is an allosteric modulator of muscarinic M1 and M4 receptors [50]. The use of clozapine is associated also with sedation, as a result of antagonism to histamine receptors, and side effects from autonomous system, including hypotension and reflex tachycardia, which in turn result from blockade of α_1_ adrenergic receptors. Clozapine is currently used mainly in forms of schizophrenia that are resistant to treatment with other drugs, which means that no satisfactory response has been achieved with at least two other drugs. The main limitation of clozapine is its tendency to cause agranulocytosis, which may lead to death. FDA suggests patients to control the number of white blood cells every week during first six months of treatment (it is the period of the highest risk of agranulocytosis). Afterwards, the number of leukocytes should be controlled every two weeks [108].

Olanzapine is a chemical analog of clozapine with similar pharmacological properties. However, contrary to clozapine, olanzapine causes fewer autonomic side effects and its use is not associated with a risk of agranulocytosis. Affinity of olanzapine to serotonin 5-HT_2_ receptors is approximately two times higher than to dopamine D_2_ receptors. It also blocks D_3_ and D_4_ receptors. Antagonism to histamine, muscarinic and α_1_-adrenoreceptors is weaker than in the case of clozapine. Clinical trials showed that efficacy of olanzapine in reducing positive symptoms is comparable with haloperidol, but is much more effective against negative symptoms and causes fewer side effects than haloperidol. The most frequent side effects of olanzapine are sedation and weight gain.

Quetiapine belongs to antipsychotics, which, besides schizophrenia, are used in treating bipolar disorders and major depressive disorders. Its off-label application includes insomnia, Tourette syndrome or anxiety disorders. Quetiapine acts as a dopamine D_1_, dopamine D_2_ and serotonin 5-HT_2_ receptors antagonist. Quetiapine is also a 5HT_1a_ receptor partial agonist. Antagonism to α_1_ adrenergic and histamine H_1_ receptor manifests in occurrence of side effects such as sedation and orthostatic hypotension.

Risperidone is the first novel atypical antipsychotic. It was introduced to the market at the beginning of the 1990s, twenty years after introduction of clozapine [113]. It is a benzisoxazole derivative (Figure 1). Its pharmacological profile is reminiscent of properties of olanzapine, except that risperidone is thought to cause sedation less frequent and orthostatic hypotension more often than olanzapine. Therapeutic effect of risperidone results from antagonism to both D_2_ and 5-HT_2A_ receptors with 5-HT_2A_ antagonism significantly stronger than the D_2_ antagonism. Moreover, this drug also causes α_1_ adrenergic and histamine receptor blockade. Its anticholinergic effects are negligible. In some patients, risperidone may elevate the level of prolactin and cause arrhythmia. The following other adverse effects may occur during treatment with risperidone: insomnia, restlessness, anxiety, headaches, agitation, EPS, headache, rhinitis, sedation, somnia, fatigue, ocular disturbances, orthostatic dizziness, palpitations, weight gain, diminished sexual desire, erectile and ejaculatory dysfunction, orthostatic dysregulation, reflex tachycardia, gastrointestinal complaints, nausea, rash, galactorrhea and amenorrhea [113,114]. Risperidone is efficient not only in treating positive symptoms but also negative and cognitive disturbances as well as has some anti-depressant properties which makes it one of the most commonly prescribed antipsychotics [113]. The active metabolite of risperidone is paliperidone, which acts on the same range of receptors. It is used in treating schizophrenia, as well as mania and, in lower doses, in bipolar disorder. Paliperidone comes in formulations of extended release, what allows to administer the drug once per day [108].

Ziprasidone, another second-generation antipsychotic, acts as antagonist of dopamine D_2_ and serotonin 5-HT_2A_ receptors, partial agonist of 5-HT_1A_ receptor, and partial antagonist of 5-HT_2C_ and 5-HT_1D_ receptors. Besides schizophrenia it is used in acute mania or bipolar disorders [115].

Molindone acts mainly by affecting dopamine transmission in the brain. It is an atypical antipsychotic with unusual profile of pharmacological properties. It rarely causes sedation and autonomic side effects but is thought to lead to extrapyramidal side effects more frequent than other new antipsychotics, although still less frequently than classical neuroleptics. The use of molindone, contrary to other second-generation antipsychotics, rarely results in weight gain. In patients that do not tolerate or respond to other drugs, the treatment with molindone is sometimes effective [108]. 

Other second-generation antipsychotics include: lurasidone, iloperidone, asenapine, sertindole and amisulpiride. They are briefly presented below.

Lurasidone is a benzisothiazole derivative with high antagonist activity at serotonin 5-HT_2A_ and 5-HT_7_ receptors and weaker antagonism at dopamine D_2_ receptor [116]. It is also a partial agonist of serotonin 5-HT_1A_ receptor and has relatively high affinity adrebergic α_2A_ and weaker affinity to muscarinic receptors [116]. In general, lurasidone is effective and well-tolerated for treatment of schizophrenia and for acute bipolar depression. It has low probability of side effects such as weight-gain, and metabolic or cardiac abnormalities, but higher risk of akathisia in comparison to other atypicals [116].

Iloperidone is a benzoxazole derivative with high affinity to serotonin 5-HT_2A_ and dopamine D_2_ receptors [117]. Iloperidone has also high affinity to α_1_ adrenergic receptors and lower affinity to dopamine D_1_ receptors, serotonin 5-HT_1A_ receptors and histamine H_1_ receptors and negligible affinity to muscarinic receptors [117]. This drug has beneficial EPS and akathisia properties which makes it attractive choice for patients whose compliance is limited by these effects [117].

Asenapine is a dibenzoxepinopyrrole derivative and has a high affinity for the serotonin 5-HT_2A_ receptor and to a lesser extent to dopamine D_2_ receptor [118] It is an antagonist of 5-HT_2C_, H_1_ and α2-receptors. It shares a rather complex binding profile with clozapine, olanzapine and quetiapine. The main side effects of asenapine are weight gain and metabolic disorders [119].

Sertindole is an indole derivative with a high affinity for dopamine D_2_, serotonin 5-HT_2A_ and 5-HT_2C_, and α_1_ adrenergic receptors [120]. It has also some affinity for histamine H_1_ and muscarinic receptors. This drug has low probability to cause sedation and EPS and displays an acceptable metabolic profile. However, cardiac safety should be monitored during treatment with sertindole.

Amisulpiride belongs to benzamides and is a specific antagonist for dopamine D_2_ and D3 receptors [121]. It has negligible affinity to serotonin receptors and receptors typically involved in side effects of atypical antipsychotics. EPS is the most common side effect of amisulpiride.

In the past decade, first-generation antipsychotics have been essentially replaced by newer, atypical antipsychotics mainly due to better toleration of second-generation antipsychotics and their more favorable profile of side effects, especially lower risk of extrapyramidal side effects. However, the second-generation antipsychotics have severe metabolic adverse effects, in particular obesity and diabetes. Weight gain and metabolic disfunctions are common in schizophrenia patients. It is attributed to the blockade of adrenergic, cholinergic, and histaminergic postsynaptic receptors by psychotropic agents [122]. Indeed, antagonism of histamine H_1_ receptors is described as a key reason of second-generation antipsychotics-induced obesity [123]. Moreover, sedation, a common side effect of clozapine treatment, can be connected with clozapine binding to histamine receptors in the CNS [124]. Type 2 diabetes mellitus is their most often reported output [125]. Metabolic side effects are mainly associated with clozapine and olanzapine while data are mixed for risperidone and quetiapine [125]. For other second-generation antipsychotics, there are only few studies which examined their metabolic effects so it is difficult to draw conclusions [125].

### 3.3. Third-Generation Antipsychotics

**Figure 2:**
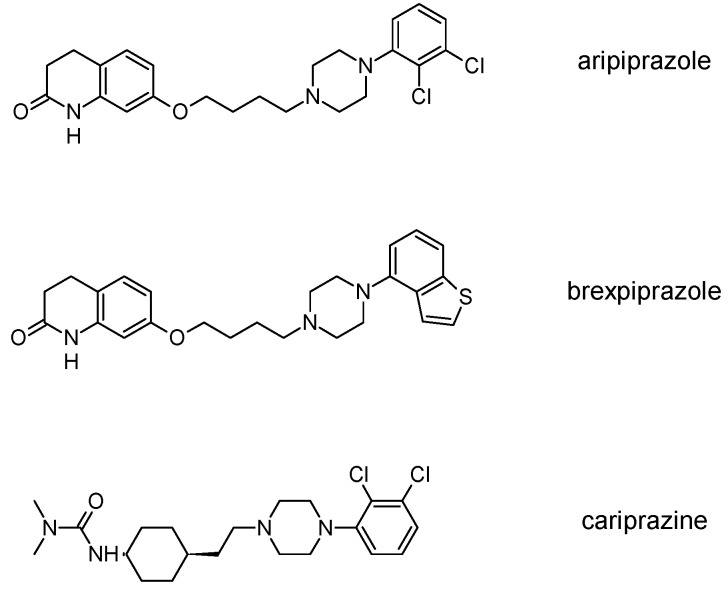
Antipsychotic drugs categorized as the third generation and their structures.

The newest group of antipsychotic drugs, described as the third generation, consists of aripiprazole, brexpiprazole and cariprazine (Figure 2). That group has been individualized on the grounds of their mechanism of action on dopamine receptors. Unlike other neuroleptics, third-generation drugs are not dopamine D_2_ receptor antagonists but D_2_ partial agonists. The D_2_ receptor partial agonist properties of aripiprazole concern inhibition of cAMP accumulation through the dopamine D_2_ receptor (i.e., G_α_ signaling) [126,127] and in the presence of high extracellular concentrations of dopamine (e.g., in mesolimbic areas), compete with dopamine and result in partial antagonism leading to clinical benefits. Contrarily, when extracellular dopamine concentration is on a low level (e.g., in dopamine circuits that are involved in working memory), aripiprazole can bind to additional receptors and activate them partially. Hence, aripiprazole is termed as “dopamine stabilizer” [128,129,130]. It has also been demonstrated that aripiprazole is an antagonist in GTPγS binding assays with dopamine D_2_ receptor [127,131]. Aripiprazole also failed to activate outward potassium currents following activation of dopamine D_2_ receptor, which can indicate that it was inactive or an antagonist for Gβγ signaling through this receptor [127]. Next, aripiprazole, as another clinically efficient antipsychotic, is an antagonist of β-arrestin pathway [132]. Moreover, aripiprazole was also reported as agonist or antagonist of other GPCRs [133]. When it comes to serotonin transmission, aripiprazole exhibits partial agonistic properties to 5-HT_1A_ and 5-HT_2A_ (much weaker in the second case), which manifests in functional antagonism at this receptor. Contrary to antipsychotic drugs classified as second generation, aripiprazole displays higher affinity for dopamine D_2_ receptor than for serotonin 5-HT_2A_ receptor. Clinical use of aripiprazole includes, besides schizophrenia, bipolar disorder, major depression, obsessive-compulsive disorder, and autism. Effectiveness of treating schizophrenia with aripiprazole is comparable with haloperidol or quetiapine and slightly higher than in the case of chlorpromazine or ziprasidone. Moreover, aripiprazole is characterized by better tolerability comparing to other antipsychotics [134]. Side effects that may result from treatment with aripiprazole include mainly akathisia [135] but also weight gain, agitation, insomnia, anxiety, headache, constipation or nausea [129]. However, aripiprazole results in considerably lower weight gain and lower increase in glucose and cholesterol levels in comparison to clozapine, risperidone, and olanzapine [136]. Next, aripiprazole led to weaker EPS, less use of antiparkinsonian drugs, and less akathisia, in relation to typical antipsychotic drugs and risperidone [136].

Brexpiprazole, approved by FDA in 2015, acts as partial agonist to dopamine D_2_, D_3_ and serotonin 5-HT_1A_ receptors, and exhibits also antagonist properties to 5-HT_2A_, 5-HT_2B_ and 5-HT_7_ receptors. Its pharmacological profile is very similar to that of aripiprazole. Brexpirazole and aripiprazle are considerably different in potencies at many receptors. Their antipsychotic efficacy is comparable but brexpiprazole causes less akathisia, EPS and activation [137]. Moreover, brexpiprazole has precognitive properties [138] in contrast to aripiprazole [139]. Brexpiprazole, alone or in combination with escitalopram, facilitates prefrontal glutamatergic transmission via a dopamine D_1_ receptor-dependent mechanism [140]. The drug is used in the treatment of schizophrenia and as an adjunct in major depressive disorder (e.g., in combination with fluoxentine [141,142]). The side effects that may result from using brexpiprazol include akathisia, weight gain, infections of upper respiratory tract, somnolence, headache and nasopharyngitis [143].

Approval of cariprazine, as well as brexpiprazole, took place in 2015. Similar to other antipsychotics from third generation, cariprazine is dopamine D_2_, D_3_ and serotonin 5-HT_1A_ receptors partial agonist. However, its affinity for dopamine D_3_ receptor is approximately ten times higher than for D_2_ receptors. Cariprazine can be considered a biased agonist at dopamine receptors, with antagonist or partial agonist activity depending on the signaling pathways linked to D_2_/D_3_ receptors [144]. Mean half-life for cariprazine is 2–5 days over a dose range of 1.5–12.5 mg [145]. Cariprazine has two clinically significant metabolites, desmethyl-cariprazine and didesmethyl-cariprazine, the latter having a longer half-life than cariprazine [145]. The clinical use of cariprazine includes schizophrenia and manic or mixed episodes associated with bipolar disorder. In particular, cariprazine can be used for the treatment of schizophrenia patients with dominant negative symptoms, a typically difficult to treat group of patients [146]. The treatment with cariprazine may lead to occurrence of side effects, such as sedation, akathisia, weight gain, nausea, constipation, anxiety, and dizziness [147]. Metabolic side effects of cariprazine are however negligible [148]. In summary, a potential for treatment negative symptoms, anti-abuse potential, and a long half-life make cariprazine a promising drug against schizophrenia [149]. 

Introduction of newer antipsychotic drugs to the clinical practice has contributed chiefly to lowering extrapyramidal side effects. Patients are less likely to suffer from akathisia, dystonias, parkinsonian symptoms or tardive dyskinesia than in the case of treatment with first generation antipsychotics. However, advantage of atypical over typical drugs when it comes to effectiveness is still discussed. Some patients respond better to newer antipsychotics and in others older drugs are more effective. This issue requires further research, which is problematic, considering schizophrenia as a chronic disease and the fact that the percentage of hospitalization of schizophrenic patients is low. It should be stressed, however, that novel compounds acting on dopaminergic system are still under investigation for the treatment of schizophrenia. Compounds such as BL 1020, ITI-007, and JNJ-37822681 have been thoroughly studied, and compounds such as L-THP, Lu AF35700, S33138, and SB-773812 are under vigorous investigation [150].

## 4. Targeting Novel GPCR Signaling Mechanisms in Schizophrenia

### 4.1. GPCRs and Their Novel Signaling Mechanisms as Drug Targets

According to the classical ternary complex model, the signaling through GPCRs can be illustrated as the coaction of three main players: a receptor, an agonist and a G protein. However, more and more experimental and computational studies support the view that GPCR functioning can be much more complex than depicted by the ternary complex model.

The discovery that a specific receptor can couple to more than one G protein type and, besides that, that GPCRs could trigger G protein-independent pathways, stimulated a more nuanced characterization of GPCR ligands. Indeed, there are ligands termed biased agonists that are capable of preferentially activating one receptor-associated pathway over another [151]. This has been connected with the existence of multiple receptor states, with different propensities to couple to G proteins or other signaling partners, and which can be differentially affected by functionally selected ligands. This complex receptor modulation, which has been termed functional selectivity [10,11], has opened a new avenue for the interrogation of specific GPCR-activated pathways and their impact on health and disease, as well as for the subsequent detection of pathway-selective drugs with a refined mechanism of action [152]. Characterization of the significance of particular pathways associated with a given receptor can provide insight into the optimal functional selectivity profile for the treatment of a particular disease. In particular, targeting β-arrestin signaling pathways can be promising in the case of antipsychotics (see below), however it should be avoided in the case of many other drugs, e.g., antinociceptive compounds targeting the µ opioid receptor [153].

At present, another hot topic in GPCR-oriented drug discovery is the design of allosteric modulators instead of orthosteric ligands [154]. Allosteric ligands possess the ability to modulate GPCR function by binding to receptor regions away from the orthosteric binding site. Allosteric modulators usually bind to receptor areas with a low degree of conservation between GPCR subtypes [155]. This binding specificity could also be the basis for the design of more selective drugs. Additionally, the fact that allosteric modulators can function together with ligands interacting at the orthosteric binding site makes drugs exploiting this phenomenon especially useful when treatment can be achieved by enhancing or decreasing an endogenous signal. Such an approach may make it possible to solve the problem of drug dependence, overdose risk and other adverse effects linked with classical orthosteric drugs. The allosteric mode of action brings several advantages, e.g., a ceiling effect preventing overdosing, high receptor selectivity, and even activation pathway selectivity which may in consequence lead to safer and more efficient drugs [15].

In addition, the growing body of experimental (cross-linking experiments, BRET and FRET studies) and computational (coarse-grained molecular dynamics simulations) reports suggesting negative and positive cooperativity between receptors, has paved the way to the concept that GPCRs can oligomerize [19,20]. Both homo- and heterodimerization was reported for numerous GPCRs and the resulting protein complexes were in some cases linked to particular functional outcomes. Thus, GPCR dimers, oligomers and receptor mosaics are now considered promising drug targets, which, due to their restricted tissue distribution, can result in tissue-specific drugs. Despite a growing number of functional interactions between dimers, drug discovery targeting GPCR complexes remains a challenge. Thus, a better description of the structural aspects of GPCR oligomerization and of its effect on signaling, accompanied by the developing of original treatment strategies, seems essential for further exploration of this mechanism of GPCR signaling [156]. 

### 4.2. Targeting GPCR Signaling Complexity in Schizophrenia

The GPCR signaling mechanisms described above have been considered as potential drug targets for novel antipsychotics. The most commonly exploited approach is intentional ligand promiscuity for multi-target drugs typical for second- and, to a lesser extent, first-generation antipsychotics which requires separation of the drug targets from the off-targets [157]. The treatment of complex diseases, such as Alzheimer’s disease, Parkinson’s disease, cancer or schizophrenia, which involve multiple receptors and enzymes in their pathomechanisms, require looking for potential drugs which satisfy the criteria of many pharmacophores, oppositely to acting on a single molecular target. It should be emphasized in schizophrenia and other complex psychiatric disorders, selective single-target drugs have a very limited efficacy. Clozapine with a nanomolar affinity to several aminergic GPCRs is efficient against drug-resistant schizophrenia and reflects the molecular pathomechanism of this disease, involving cross-talk of many neurotransmitter systems (in particular, dopaminergic, serotonergic, adrenergic and glutamatergic). The new paradigm in medicinal chemistry is to look for substances that act on several molecular targets simultaneously. To accomplish that, it is essential to find structural features which combine important classes of drug targets, leading to molecules with desired selectivity profiles. Although recently implement antipsychotics (e.g., cariprazine and brexpiprazole) are the third-generation drugs, efforts are still made to design new multi-target ligands which can be developed into second generation antipsychotics [29,158]. 

The next approach that is currently under extensive investigation is biased signaling and functional selectivity. D_2_ receptors couple to Gα_i/o_ subunits, which leads to a number of signaling events through the release/rearrangement of G proteins, involving inhibition/sensitization of adenylyl cyclase, G_βγ_ potentiation of AC2, and ERK activation as well as β-arrestin pathway [159,160]. Latest reports allow concluding that selective modulation of signaling pathways downstream of the D_2_ receptor can lead to development of more efficient and safer antipsychotics [161]. In particular, blockade of β-arrestin pathway can be considered a common feature of antipsychotics that exhibit either antagonist or partial agonist activity through Gα_i/o_-cAMP pathways [161]. This supposes that β-arrestin-biased D_2_ antagonists can display unique antipsychotic profiles [161]. Contrary to that, a study with analogs of aripiprazole concluded that D_2_ ligands with Gα_i/o_ antagonist and β-arrestin agonist activity can display antipsychotic properties with diminished extrapyramidal side effects in animal model [7]. Several series of biased D_2_ agonists have been reported [7,162,163] as underlying ligand and receptor structural features necessary for biased signaling. Moreover, as has already been mentioned, serotonin 5-HT_2A_ receptor functional selectivity can be important for the activity of antipsychotics as it was reported for clozapine [112,164].

**Figure 3:**
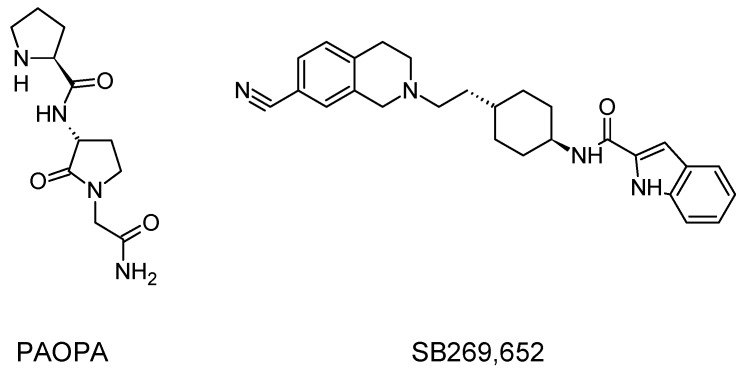
Allosteric modulators of dopamine D_2_ receptor with possible application for the treatment of schizophrenia.

Allosteric modulation of D_2_ receptor as a mechanism for novel antipsychotics has been not sufficiently studied. Only a peptidomimetic PAOPA (Figure 3), a positive allosteric modulator of D_2_ receptor, has been proven to be successful in animal models of schizophrenia. Few negative allosteric modulators are known, e.g. SB269,652, homocysteine and analogs, see Figure 3 and their antipsychotic potential needs to be evaluated. A clear finding of antagonist allosteric actions on D_2_ receptor has been demonstrated for homocysteine and analogs, which supports the possibility that homocysteine analogs can be developed into an efficient antipsychotic drug, since they reduce in vitro dopamine binding. Allosteric compound SB269,652 seems to act on D_2_ receptor dimer [165], similarly as bivalent D_2_ receptor ligands based on agonist or antagonist structures [166,167], which are however not drug-like due to their high molecular weight and are pharmacological tools rather than potential drugs. At present, no dimer-specific monovalent ligands are known for D2 receptor homodimers or heterodimers of this receptor and no ligands inducing or disrupting dimerization are known. 

In addition to allosteric modulators of dopamine D_2_ receptor, positive allosteric modulators of metabotropic glutamate receptors are nowadays studied as possible treatment for schizophrenia [168]. In particular, PAMs of mGluR1, mGluR2, mGluR3 and mGluR5 are promising. Furthermore, PAMs of muscarinic receptors, e.g., M_1_ and M_4_ receptor could be also useful [168].

Targeting D_2_-receptor-containing dimers is a new and yet unexploited strategy for the treatment of schizophrenia. Among heterodimers formed by the D_2_ receptor, adenosine A_2A_–D_2_ and serotonin 5-HT_2A_–D_2_ heterodimers in particular are implicated in the pathomechanism of schizophrenia [169]. A possible biochemical alteration in schizophrenia might not only be D_2_ receptor sensitization with elevated D_2_ receptor signaling, but also decreased A_2A_ receptor activity or interruption of A_2A_–D_2_ receptor interactions because of the existence of abnormal A_2A_–D_2_ receptor heteromers or by their disappearance [170]. Observations supporting the presence of A_2A_–D_2_ heteromers with antagonistic A_2A_–D_2_ interactions in the ventral striatopallidal GABA pathway suggested the use of A_2A_ receptor agonists as a strategy for the treatment of schizophrenia [171]. The antipsychotic potential of A_2A_ receptor agonists is underlined through behavioral analysis in animal models of schizophrenia in which they demonstrated an atypical antipsychotic profile [171]. Other classes of potential antipsychotics acting through the A_2A_–D_2_ receptor heteromer may be the heteromer selective D_2_ receptor orthosteric or allosteric antagonists or compounds promoting ligand-induced dimerization. The possible involvement of the D_2_–5-HT_2A_ heterodimer in schizophrenia was proposed by studies on the hallucinogenic 5-HT_2A_ agonists showing that they probably induce a conformational state of the 5HT_2A_ protomer different from the one produced by serotonin, leading to facilitating allosteric interactions with the D_2_ partner [171]. This increase in the D_2_ receptor signaling in the D_2_–5-HT_2A_ heterodimer in the nucleus accumbens could be the basis for their psychotic actions [171]. This fact can also supply a better description of the molecular mechanism of the therapeutic effects of the second-generation antipsychotics, such as risperidone and clozapine, which block the 5-HT_2A_ receptor with higher potency than the D_2_ receptor. It can be postulated that, in some cases of schizophrenia, this pathological facilitating interaction between 5-HT_2A_ and D_2_ receptors has developed in the 5-HT_2A_–D_2_ heterodimer leading to increased D_2_ protomer recognition and signaling [171]. Interestingly, 5-HT_2A_ or D_2_ receptor antagonists acting selectively on this heterodimer or compounds disrupting the heterodimer interface might be a novel strategy to treat schizophrenia. Regarding other dimers with possible involvement in the pathomechanism of schizophrenia, mGluR2–5-HT_2A_ heteromer should be mentioned [172] This complex displays unique signaling when interacting with hallucinogenic drugs and activation of mGluR2 cancels hallucinogenic signaling and related behavioral responses. In postmortem studies of human brains from untreated schizophrenic subjects, the 5-HT_2A_ receptor is up-regulated and the mGluR2 is down-regulated which can predispose to psychosis. 

## 5. Other Non-Classical Approaches for the Treatment of Schizophrenia 

The limitations of current antipsychotics are supplemented by reports about involvement of neurtotransmitter systems other than the dopaminergic system, especially glutamate neurotransmission that contributes to the pathomechanism of schizophrenia. Thus, new drug targets resulting in drugs with novel mechanisms of action have been proposed. In particular, glutamate and nicotinic targets seems promising [173,174]. Selective ligands for metabotropic glutamate receptor, phosphodiesterase, glycine transporter subtype 1 and the alpha7 nicotinic acetylcholine receptor are considered worth further investigation [173,174]. 

Regarding metabotropic glutamate receptors, potentiation of mGluR2/3 and mGluR5 receptors can be beneficial in schizophrenia [175]. Although classical orthosteric agonists are still an option, subtype-selective allosteric ligands, including positive allosteric modulators of mGluR2 and mGluR5 can offer numerous advantages brought by allosteric mode of action. 

Phosphodiesterase (PDE) inhibitors improve neurotransmission by affecting intracellular second messenger signaling [176]. In particular, inhibitors of PDE2, PDE4, PDE5 and PDE10 seem promising for treating cognitive symptoms of schizophrenia.

Glycine transporter 1 inhibitors applied in combination with antipsychotics are effective for relieving negative symptoms of the disease. α7 nicotinic receptor agonists and positive allosteric modulators and minocycline may treat negative and cognitive symptoms. Complementary oxytocin may help to ameliorate psychotic symptoms and social cognitive deficits. Complementary erythropoietin might benefit cognitive function [174].

## 6. Conclusions and Future Prospects 

Although current concept and treatment of schizophrenia are still based on the dopaminergic hypothesis of the disease, novel approaches involving new signaling mechanisms on classical drug targets or completely new targets emerge. Schizophrenia is a complex multi-factor disease and according to the current knowledge it does not seem very probable that all symptoms of the disease can be treated with a single-target drug. Indeed, clozapine, which is used to treat resistant schizophrenia has a nanomolar affinity to a dozen of aminergic GPCRs. Searching for multi-target drugs beyond aminergic GPCRs may be a promising strategy to design better antipsychotics. Moreover, novel signaling mechanisms of GPCRs can lead to safer drugs with fewer side effects, e.g., allosteric modulators or biased ligands or even with tissue specificity (dimer-selective ligands). Current efforts in drug design against schizophrenia focus on searching for compounds to treat negative symptoms and to improve cognitive deficits as well as searching for compounds that are better tolerated by patients who often need life-lasting treatment.

In summary, after over a century of schizophrenia treatment, significant progress has been achieved, starting from lobotomy operations, through discovery of chlorpromazine to current second- and third-generation antipsychotics. Novel approaches, following new findings in the disease mechanisms, will finally result in new generations of drugs.

## Figures and Tables

**Table 1:** Classification of first-generation antipsychotic drugs: examples and their chemical structures.

Group of Antipsychotic Drugs			Drug Examples
**Phenothiazines** 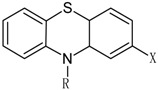	**Side chain**	**Aliphatic**	Chlorpromazine X = Cl; R = 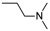
			Levomepromazine X = OCH_3_; R = 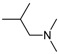
			Promazine X = H; R = 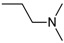
			Triflupromazine X = CF_3_; R = 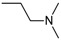
		**Piperidine**	Mesoridazine X = SOCH_3_; R = 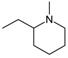
			Pericyazine X = CN; R = 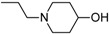
			Pipotiazine X = SO_2_N(CH_3_)_2_; R = 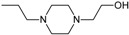
			Thioridazine X = SCH_3_; R = 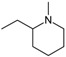
		**Piperazine**	Fluphenazine X = CF_3_; R = 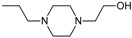
			Perphenazine X = Cl; R = 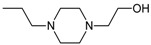
			Prochlorperazine X = Cl; R = 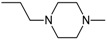
			Trifluoperazine X = CF_3_; R = 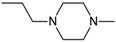
**Butyrophenones** 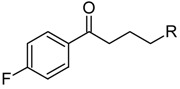			Benperidol 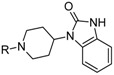
			Droperidol 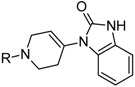
			Haloperidol 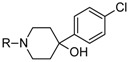
**Thioxanthenes** 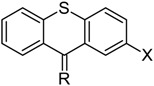			Clopenthixol X = Cl; R = 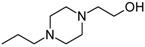 mixture of *cis* and *trans* isomers
			Flupenthixol X = CF_3_; R = 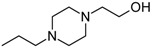 mixture of *cis* and *trans* isomers
			Thiothixene X = SO_2_N(CH_3_)_2_; R = 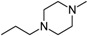 *cis* isomer
			Zuclopenthixol X = Cl; R = 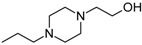 *cis* isomer
**Dihydroindolones**			Molindone 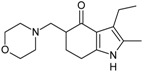
**Dibenzepines** 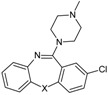			Clotiapine X = S
			Loxapine X = O
**Diphenylbutylpiperidines** 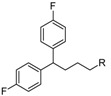			Fluspirilene 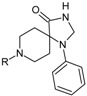
			Pimozide